# Phenotypic and genomic modeling of lactation curves: A longitudinal perspective*

**DOI:** 10.3168/jdsc.2023-0460

**Published:** 2024-02-01

**Authors:** Hinayah Rojas de Oliveira, Gabriel S. Campos, Sirlene F. Lazaro, Janusz Jamrozik, Alan Schinckel, Luiz F. Brito

**Affiliations:** 1 Department of Animal Sciences, Purdue University, West Lafayette, IN 47907; 2 Centre for Genetic Improvement of Livestock (CGIL), Department of Animal Biosciences, University of Guelph, Guelph, ON, N1G 2W1 Canada; 3 Lactanet Canada, Guelph, ON, N1K 1E5 Canada

## Abstract

Lactation curves, which describe the production pattern of milk-related traits over time, provide insightful information about individual cow health, resilience, and milk production efficiency. Key functional traits can be derived through lactation curve modeling, such as lactation peak and persistency. Furthermore, novel traits such as resilience indicators can be derived based on the variability of the deviations of observed milk yield from the expected lactation curve fitted for each animal. Lactation curve parameters are heritable, indicating that one can modify the average lactation curve of a population through selective breeding. Various statistical methods can be used for modeling longitudinal traits. Among them, the use of random regression models enables a more flexible and robust modeling of lactation curves compared with traditional models used to evaluate accumulated milk 305-d yield, as they enable the estimation of both genetic and environmental effects affecting milk production traits over time. In this symposium review, we discuss the importance of evaluating lactation curves from a longitudinal perspective and various statistical and mathematical models used to analyze longitudinal data. We also highlighted the key factors that influence milk production over time, and the potential applications of longitudinal analyses of lactation curves in improving animal health, resilience, and milk production efficiency. Overall, analyzing the longitudinal nature of milk yield will continue to play a crucial role in improving the production efficiency and sustainability of the dairy industry, and the methods and models developed can be easily translated to other longitudinal traits.

## Graphical Abstract

**Figure:**
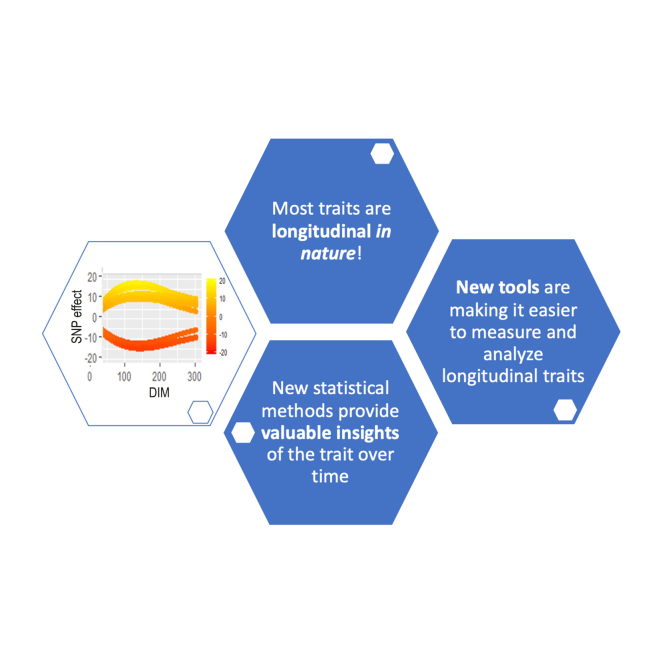

Lactation curve mathematical modeling is an approach used to describe the production pattern of milk-related traits over time. In general, dairy animals exhibit a relatively high rate of increase in milk production from parturition until reaching the peak milk yield (4–8 wk), after which daily milk yield is usually maintained at a similar level for a certain period (depending on the lactation persistency of the animal), and subsequently declines until the animal is dried off (Strucken et al., 2015; Cole et al., 2023). Modeling and comprehensively understanding lactation curve patterns can provide valuable insights into milk yield variability, enable the estimation of total milk production from incomplete milk records, facilitate forecasting of herd performance on a monthly or individual cow basis, support more efficient feeding management strategies, and contribute to identifying health issues on individual animals. To this end, researchers have used both phenotypic and genomic modeling approaches to study lactation curves (e.g., Angeles-Hernandez et al., 2021; Ben Abdelkrim et al., 2021), but limited information is currently being used in the dairy industry and translated to dairy farmers.

In this context, dairy breeding programs have focused on practical measurements derived from the lactation curves, such as accumulated milk production, lactation persistency, and lactation length. This is mainly due to the greater complexity of statistical models that take into account repeated records (e.g., random regression model, **RRM**; Oliveira et al., 2019a) in comparison to single-trait animal models (Schaeffer and Jamrozik, 2008; Oliveira et al., 2019a). Moreover, a patent applied by Cornell University in 1993 forestalled, to some extent, the use of RRM in the United States (Powell and Norman, 2006). However, this patent has now expired, which might potentially increase the use of RRM in official US evaluations in the near future.

A comprehensive review of the advantages of the use of RRM can be found in Schaeffer and Jamrozik (2008) and Oliveira et al. (2019a). In summary, using RRM to analyze repeated records allows us to account for the dynamic nature of lactation curves, by fitting individual covariance functions to model both random genetic and environmental effects over time (Schaeffer, 2004; Oliveira et al., 2019a). Thus, analyzing the complete lactation curve information holds great potential in assessing metabolic and reproductive disorders in dairy cattle, as most disorders are associated with negative energy balance (expressed in milk production) that some cows undergo at the beginning of lactation (e.g., Pryce et al., 2016). Moreover, milk yield is largely affected by environmental disturbances such as heat stress, feeding availability and quality, and diseases. Therefore, variability metrics in the expected and produced daily milk yield records across lactation allow the derivation of resilience indicator traits (e.g., Poppe et al., 2021; Wang et al., 2022; Chen et al., 2023). Derived traits include natural log-transformed variance, lag-1 autocorrelation of daily deviations in milk yield from an expected lactation curve, and milk losses (Wang et al., 2022).

By leveraging large-scale and genome-wide genomic information, genetic variants associated with milk production traits at specific time points can be identified, which contributes to improving the understanding of the underlying biological mechanisms influencing lactation curves. In this symposium review, we first summarized the main statistical models used to analyze longitudinal data in dairy cattle and described the primary factors contributing to postponing the use of RRM in official genomic evaluations. Furthermore, we highlighted the recent advances and applications of longitudinal analyses of lactation curves to improve animal health, resilience, and milk production efficiency, to complement and update our last invited review paper published in the *Journal of Dairy Science* (i.e., Oliveira et al., 2019a).

Repeatability, autoregressive, and multiple-trait models have been used to analyze test-day milk yield records, but they all have some limitations. For instance, the genetic correlation between records is considered to be equal to 1 in repeatability models, which is biologically unrealistic (Ptak and Schaeffer, 1993). Using autoregressive models has the advantage of capturing temporal dependencies and relationships over time; however, the structure of the dependence usually needs to be assumed before the analyses (Sawalha et al., 2005). On the other hand, evaluating each record as a different trait (i.e., multiple-trait models) has the advantage of not making prior assumptions, but it can lead to over-parametrization problems and high computational demand (Misztal et al., 2000). Therefore, these methods for modeling longitudinal traits usually fail to consider the individual shape of the lactation curves.

Different linear and nonlinear functions have also been developed for modeling lactation curves in dairy species. Over 30 linear or nonlinear models containing 3, 4, 5, or more parameters, along with different polynomial functions, have been reported in the literature. The most common models used to describe the shape and parameters of the lactation curve are Wood (Wood, 1967), Wilmink (Wilmink, 1987), and Ali and Schaeffer (Ali and Schaeffer, 1987). Modeling lactation curves using these mathematical functions has been widely used over the past decades in several dairy species (e.g., Jenko et al., 2017; Lázaro et al., 2021).

The main challenge of using linear and nonlinear functions in official genetic evaluations is to ensure an accurate fit of the model to the observed data. Factors such as genetic differences, environmental influences, and management practices can make it more difficult. Additionally, outliers, missing data, and differences in milk yield recording protocols can affect the model's convergence and estimability. Selecting an appropriate model structure that adequately represents the diversity of lactation curves observed within and across breeds or populations is also a challenge. Continuous research and refinement of lactation curve models, along with robust statistical methods, are necessary to address these challenges and improve the accuracy of official genetic and genomic evaluations. To avoid some of these challenges, RRM based on linear functions are the most widely used models for the genetic evaluation of milk traits due to their flexibility and accuracy (Oliveira et al., 2016). The most used linear functions are orthogonal polynomials, such as the Legendre orthogonal polynomials (Kirkpatrick et al., 1990) and smoothing spline functions like B-spline (White et al., 1999; Meyer, 2005).

Regardless of the function or polynomial used, RRM consider the covariance structure pattern among repeated measures by enabling the fit of individual covariance functions to model both random genetic and environmental effects over time (Schaeffer, 2004; Oliveira et al., 2019a). By accounting for individual variations and capturing the nonlinear nature of lactation curves, RRM provide a comprehensive description of the complex factors shaping milk production trajectories. The versatility and adaptability of RRM make them a valuable tool for modeling longitudinal traits.

It is well known that genomic prediction increases the accuracy of genomic estimated breeding values (**GEBV**) and genomic selection contributes to decreasing generation interval, especially in dairy cattle. Consequently, genomically assisted breeding accelerates the genetic progress for important economically important traits, such as milk yield and composition. However, studies focusing on genomic prediction of the lactation curve are still scarce in the literature in comparison to studies fitting accumulated milk production. This is mainly related to 3 factors: (1) the gains in breeding value accuracy due to use of test-day records appears to be lower than the improvements in accuracy due to the inclusion of genomic information when using the 2-step genomic BLUP (**GBLUP**) approach (Oliveira et al., 2019b); (2) there is an increased computational demand needed for the evaluations using genomic RRM (Oliveira et al., 2019c); and (3) there is still a need to summarize all the information in a single or few estimates for practical selection decisions.

Recent results have shown that the use of the single-step GBLUP (**ssGBLUP**) approach based on RRM yielded more reliable and less biased GEBVs in comparison to the use of pedigree-based parent average (**PA**) generated by RRM, as observed in a large dairy cattle study including over 70 million phenotypes and 20,663 genotyped animals (Oliveira et al., 2019c). A large dataset with 629,769 test-day records of Canadian Holstein milk infrared spectra was also used to predict GEBVs for milk fatty acid (**FA**) traits based on single-trait RRM models (Freitas et al., 2020). Later, GEBVs for milk production and milk FA traits in the first lactation of Walloon Holstein cattle were predicted based on a 2-trait RRM and ssGBLUP with gains in reliability in comparison to PA predicted using RRM (Paiva et al., 2022). Single-step genomic RRM have also been recently used for genomic studies in other dairy species. For instance, Lázaro et al. (2021) used genomic RRM to predict various milk-related traits (i.e., fat and protein yields and SCS) in Murrah water buffaloes. The authors concluded that the genetic evaluation based on the ssGBLUP RRM was easier to implement and yielded less biased breeding values compared with the pedigree-based BLUP using RRM.

The RRM have also been used to predict GEBVs for different resilience indicator traits in Holstein cattle (e.g., Poppe et al., 2021; Chen et al., 2023). In this context, third- and fourth-order Legendre orthogonal polynomials, as well as different parametric functions, were fitted to obtain the expected daily milk yield for each animal, which were used to derive the resilience indicator traits. The RRM have also been used for genetically modeling traits derived from automated milking systems (milking robots), including milk yield, SCS, milk electrical conductivity, milking efficiency, average milk flow rate, maximum milk flow rate, and milking time (e.g., Pedrosa et al., 2023). Consequently, changes in genetic parameters across the lactation curve were observed for all traits, which has important breeding and management implications. A genomic RRM has also been used for evaluating blood BHB predicted from milk infrared spectra as an indicator of ketosis in Holstein cattle, which showed higher heritability estimates for the early and lower for the later lactation periods (Lou et al., 2022).

The cow milk production changes due to responses to an increase in temperature-humidity index (**THI**) as an indicator of heat tolerance have also been evaluated using genomic RRM (e.g., Bohlouli et al., 2021). In this context, genomic reaction norm models were applied to estimate genetic parameters using a single-step approach for various milk-related traits in dependency of THI at different lactation stages (genotype by environment per lactation stage). Consequently, GEBVs were predicted under both thermoneutral and heat stress conditions (Bohlouli et al., 2021). A complete review about the genomic tools and statistical models (including RRM) that are currently available to identify climate-resilient dairy cows can be found in Silpa et al. (2021).

Many countries that are members of Interbull have implemented RRM in their national dairy cattle genetic evaluations (Interbull, 2018). The Interbull multiple across-country evaluations (MACE) provide breeding values for total lactation yields for internationally used bulls to the national breeding organizations of participant countries. Based on this, Boerner et al. (2023) proposed a method to integrate Interbull breeding values as pseudo-data points for milk, fat, and protein yield into the domestic single-step RRM in Australian Red Dairy breeds. They concluded that the pseudo-data approach aligns well with Interbull and postintegration breeding values. According to the authors, the method is easy to implement because it is unnecessary to manipulate the right-hand side or a particular covariance structure between individuals in the mixed model equations (Boerner et al., 2023).

As the number of genotyped animals has increased around the world, new methods need to be developed to overcome the computational demands needed to evaluate complex models, such as RRM. Nowadays, the North American Holstein genetic evaluation contains more than 8 million genotyped individuals (https://uscdcb.com/database-stats/), which has a high computing cost and is not feasible to obtain a direct inversion of a genomic relationship matrix (**G**) of this dimension. In this context, Misztal et al. (2014) proposed an algorithm known as the algorithm for proven and young animals (APY), which calculates a sparse inverse of the **G** matrix based on recursion methods. Other methods have been implemented where the **G^−1^** is replaced by a computational formula based on the **G** structure via Woodbury matrix identity (**ssGTBLUP**; Strandén et al., 2019). Mäntysaari et al. (2020) illustrated the computing costs of a RRM using 178,000 genotyped animals and 8.3 million animals with records in the ssGTBLUP. The basic RRM was the same used in the official Nordic Holstein production trait evaluation (Lidauer et al., 2015), with 9 traits and 42 random regression coefficients for each animal, and it took 12.5 h to complete.

Finally, recent advancements in phenomics are expected to improve the efficiency of genomic selection. High-throughput phenotyping technologies are now a crucial component of modern livestock systems because these tools can deliver real-time, accurate, and noninvasive information at the individual animal level (Bresolin and Dórea, 2020). In relation to milk production, mid-infrared spectrometry (**MIR**) has been commonly used worldwide to predict records for several traits (e.g., De Marchi et al., 2014; Grelet et al., 2020; Shadpour et al., 2022). Thus, the vast amount of data generated by sensors and automated devices, with possibly thousands of time points, presents new opportunities to derive novel phenotypes and consequently apply RRM for genetic and genomic evaluations of the derived traits. A summary of the studies mentioned in this section is included in the Table 1.

**Table 1:** Summary of recent findings related to genomic evaluation of lactation curves^1^

Reference	Model	Traits	Main conclusions
Oliveira et al. (2019c)	Multitrait RRM	Milk, fat, and protein yields, and SCS	Less inflated GEBVs were obtained using ssGBLUP RRM compared with traditional BLUP RRM.
Freitas et al. (2020)	Single-trait RRM	Milk fatty acid traits	Moderately to highly accurate GEBVs can be obtained for daily points throughout lactation using ssGBLUP RRM.
Lázaro et al. (2021)	Single and multitrait RRM	Milk, fat, and protein yields, and SCS	Genetic evaluation is easier to implement with ssGBLUP RRM and yield less biased EBV compared with pedigree-based BLUP RRM.
Bohlouli et al. (2021)	Single-trait RRM (reaction norm model)	Milk production and milk fatty acid traits	Results indicate moderately accurate genomic predictions for milk traits in extreme THI classes.
Paiva et al. (2022)	Single-trait RRM	Milk yield, fat and protein contents, and fatty acid traits	The third-order Legendre polynomials seem to be most parsimonious and sufficient to describe milk production and fatty acid traits using ssGBLUP RRM.
Lou et al. (2022)	Repeatability and single-trait RRM	BHB concentration in blood	Selecting for lower predicted BHB levels in early lactation could reduce ketosis incidence and potentially enhance reproductive and longevity performance.
Chen et al. (2023)	Multitrait RRM	Resilience indicator traits	Genomic-based genetic parameters were estimated for different resilience indicators derived from daily milk yield.
Boerner et al. (2023)	Multitrait across-country RRM	Milk and fat yield traits	The methodology and results presented suggest an approach for integrating Interbull breeding values into a domestic single-step RRM.

1 RRM = random regression model; GEBV = genomic estimated breeding values; ssGBLUP = single-step genomic BLUP; THI = temperature-humidity index.

The physiological changes observed in animals during the lactation period are influenced by variations in time-dependent gene expression. Significant changes in additive genetic variance and heritability over the lactation period have been reported (Lázaro et al., 2021; Paiva et al., 2022), suggesting differential gene expression throughout lactation. However, most GWAS analyzing longitudinal traits have still aggregated repeated records for each animal into a single estimate (e.g., Pedrosa et al., 2021; Massender et al., 2023). This conventional method limits the ability to identify genomic regions and candidate genes with substantial effects at specific time points but not an overall significant effect. Consequently, using statistical models such as RRM (which account for variability across repeated records) can substantially enhance the statistical power to detect time-dependent QTL and candidate genes.

Oliveira et al. (2019d) showed that the SNP effects and corresponding positional candidate genes were potentially associated with milk, fat, and protein yields, and SCS change over the lactation, in Ayrshire, Holstein, and Jersey Canadian breeds. The SNP solutions obtained for the RRM coefficients were converted into SNP effects over time (from 5 to 305 d in lactation) with the top 1% of SNP with the highest magnitude of SNP effect in at least 1 d in lactation considered as the most relevant. The findings from this mathematical approach corroborate with results obtained by Strucken et al. (2011), who also reported that specific genes or QTL are differently expressed depending on the lactation stage.

Freitas et al. (2020) performed a single-step GWAS aiming to identify genomic regions, candidate genes, and metabolic pathways associated with 5 groups of milk FA (i.e., short-chain, medium-chain, long-chain, saturated, and unsaturated) predicted based on MIR spectra in Holstein cattle and RRM. Various genomic regions associated with the mentioned traits were identified, but mainly with individual small effects on the total genetic variation. Regardless, the authors suggested a potential differential phenotypic expression of the candidate genes and pathways over the lactation curve. Similarly, Lázaro et al. (2021) also estimated longitudinal SNP effects and candidate genes potentially associated with time-dependent variation in milk, fat, and protein yields, as well as SCS in multiple parities of Murrah buffaloes, based on 3 lactation stages (from 5 to 70, from 71 to 150, and from 151 to 305 DIM) for each trait and parity. The authors reported evidence of differential sets of candidate genes underlying the phenotypic expression of the traits across parities.

Genome-wide associations were also recently performed to assess the heat stress response in milk FA composition in German Holsteins (Bohlouli et al., 2022). The authors used the different definitions of milk FA heat stress indicators (created based on the milk FA breeding values in response to changes in THI), in the GWAS and gene annotation analyses. The FA from 3 lactation stages were considered as different, but genetically correlated traits using a multiple-trait reaction norm model (using either the pedigree-based or genomic relationship matrix). The authors reported that genes related to milk FA might be differentially expressed under varying climatic conditions in different lactation stages.

In addition to phenotypic and genomic data, the integration of multiomic datasets, including transcriptomics, metabolomics, and epigenomics, will provide valuable insights into the regulatory mechanisms influencing gene expression over time. Unraveling the temporal dynamics of gene regulatory networks will be crucial for understanding how specific genes exert their influence on different lactation stages. By combining these multiomics datasets with the longitudinal SNP effects from GWAS, we will gain a more comprehensive understanding of the genetic architecture underlying lactation traits. This knowledge will not only enhance our understanding of lactation physiology, but it has also practical implications for breeding programs aiming to optimize milk production and quality, animal resilience, and cow health throughout the lactation period. Moreover, this integrative approach can aid in developing targeted interventions and management strategies tailored to different lactation stages, ultimately contributing to improved metabolic and reproductive health in dairy cattle. A summary of the studies mentioned in this section is included in the Table 2.

**Table 2:** Summary of recent findings related to GWAS of lactation curves

Reference	Model^1^	Traits	Main conclusions
Strucken et al. (2011)	Wood, Wilmink, and Ali and Schaeffer models	Milk, fat, and protein yields; and fat and protein contents	Estimation of candidate gene effects over the whole lactation and in different lactation stages.
Freitas et al. (2020)	Single-trait RRM	Milk fatty acid composition traits	SNP effects were estimated over time from 5 to 305 d in lactation. The variance explained by 20 adjacent SNPs ranged from 0.71% to 15.12%.
Oliveira et al. (2019d)	Multitrait RRM	Milk, fat, and protein yields	SNP effects estimated over time from 5 to 305 d in lactation help to identify a different set of candidate genes for each breed, and similar genes were found across different lactations.
Lázaro et al. (2021)	Single- and multitrait RRM	Milk, protein, and fat yields, and SCS	SNP effects estimated over time from 5 to 305 d in lactation suggest differential sets of candidate genes underlying the phenotypic expression of the traits across parities.
Pedrosa et al. (2021)	Single-trait linear mixed model	Lactation persistency (LP) and milk production (MPD) traits	A variety of genomic regions related to LP and MPD were previously associated with fertility traits, confirming the links between these 2 groups of traits.
Bohlouli et al. (2022)	Single-trait linear mixed model	Fatty acid (FA) content traits	The results indicate that genes related to FA are differentially expressed under varying climatic conditions in different lactation stages.

1 RRM = random regression model.

Overall, the knowledge and application of statistical methodologies, mathematical models, and phenotypic modeling techniques provide valuable tools for improving milk yield prediction, animal health and welfare, genetic and genomic evaluations, and decision-making in dairy farms. Furthermore, leveraging the information from the complete lactation curve facilitates the identification of cows with superior performance across various lactation stages, enabling the selection of animals with more stable and sustainable milk production patterns. As a result, the strategic use of lactation curve data in breeding programs has the potential to reduce the incidence of metabolic and reproductive disorders, ultimately enhancing the overall productivity, welfare, and longevity of dairy cattle herds. Future research should focus on refining current modeling approaches by incorporating more sophisticated statistical techniques (such as the use of machine learning and hierarchical models) and integrating new data sources (e.g., sensor technologies, multiomic datasets) to enhance the accuracy and applicability of phenotypic modeling.
